# CT-based radiomic prognostic vector (RPV) predicts survival and stromal histology in high-grade serous ovarian cancer: an external validation study

**DOI:** 10.1007/s00330-024-11267-5

**Published:** 2024-12-11

**Authors:** Georg J. Wengert, Haonan Lu, Eric O. Aboagye, Georg Langs, Nina Poetsch, Ernst Schwartz, Zsuzsanna Bagó-Horváth, Christina Fotopoulou, Stephan Polterauer, Thomas H. Helbich, Andrea G. Rockall

**Affiliations:** 1 Department of Surgery and Cancer, Faculty of Medicine, Imperial College London, London, UK; 2 Department of Biomedical Imaging and Image-guided Therapy, Medical University of Vienna, Vienna General Hospital, Vienna, Austria; 3 Computational Imaging Research Laboratory, Department of Biomedical Imaging and Image-guided Therapy, Medical University of Vienna, Vienna General Hospital, Vienna, Austria; 4 Department of Pathology and Comprehensive Cancer Center, Medical University of Vienna, Vienna General Hospital, Vienna, Austria; 5 Department of Obstetrics and Gynecology, Medical University of Vienna, Vienna General Hospital, Vienna, Austria; 6 Department of Radiology, Imperial College Healthcare NHS Trust, London, UK

**Keywords:** High-grade serous ovarian cancer, Radiomic prognostic vector, External validation, Computed tomography, Fibronectin

## Abstract

**Objectives:**

In women with high-grade serous ovarian cancer (HGSOC), a CT-based radiomic prognostic vector (RPV) predicted stromal phenotype and survival after primary surgery. The study's purpose was to fully externally validate RPV and its biological correlate.

**Materials and methods:**

In this retrospective study, ovarian masses on CT scans of HGSOC patients, who underwent primary cytoreductive surgery in an ESGO-certified Center between 2002 and 2017, were segmented for external RPV score calculation and then correlated with overall survival (OS) and progression-free survival (PFS). A subset of tissue samples subjected to fibronectin immunohistochemistry were evaluated by a gynaeco-pathologist for stromal content. Kaplan–Meier log-rank test and a Cox proportional hazards model were used for outcome analysis.

**Results:**

Among 340 women with HGSOC, 244 ovarian lesions were available for segmentation in 198 women (mean age 59.8 years, range 34–92). Median OS was 48.69 months (IQR: 27.0–102.5) and PFS was 19.3 months (IQR: 13–32.2). Using multivariate Cox analysis, poor OS was associated with RPV-high (HR 3.17; 95% CI: 1.32–7.60; *p* = 0.0099), post-operative residual disease (HR 2.04; 95% CI: 1.30–3.20; *p* = 0.0020), and FIGO stage III/IV (HR 1.79; 95% CI: 1.11–2.86; *p* = 0.016). Age did not influence OS. RPV-high tissue had higher stromal content based on fibronectin expression (mean 48.9%, SD 10.5%) compared to RPV-low cases (mean 14.9%, SD 10.5%, *p* < 0.0001). RPV score was not significantly associated with PFS.

**Conclusion:**

Patients with HGSOC and RPV-high ovarian mass on pre-operative CT had significantly worse OS following primary surgery and a higher stromal content compared to RPV-low masses, externally validating the RPV and its biological interpretation.

**Key Points:**

***Question*** *Can the performance of a previously described RPV in women with HGSOC be replicated when licenced to an external institution*?

***Findings*** *External validation of RPV among 244 ovarian lesions demonstrated that, on multivariate analysis, OS was associated with RPV, stage, and postoperative residual disease, replicating previous findings*.

***Clinical relevance*** *External validation of a radiomic tool is an essential step in translation to clinical applicability and provides the basis for prospective validation. In clinical practice, this RPV may allow more personalized decision-making for women with ovarian cancer being considered for extensive cytoreductive surgery*.

## Introduction

Medical imaging is integral to modern healthcare for the detection and staging of disease and for the planning and monitoring of oncologic treatments [1]. Advances in computational networks now allow a deeper interrogation of biomedical images, that represent the product of pathophysiologic processes and anatomic expression of a tissue [2]. Radiomic analysis is based on the extraction of imaging features, that are beyond the capacity of human perception. This may offer a unique opportunity to predict clinical parameters and support evidence-based decisions for personalized care [3]. To implement such deep-learning tools into clinical practice, robust validation is required and must include external datasets from another center [4, 5]. In ovarian cancer (OC), patient selection for primary surgery (prior to chemotherapy) can be challenging, and a majority of women present with an advanced stage [6]. Identification of women who would likely experience the best outcome from radical resection of all macroscopic diseases is important, while avoiding surgery in those unlikely to have a beneficial outcome. Improvements in detection and risk stratification are currently being attempted using genetic biomarkers [7, 8], and radiological imaging assessment [9, 10]. However, up to one-quarter of advanced OC patients relapse within twelve months of radical surgery despite complete tumor clearance, thus putting the value of surgery for those patients into question, as they may have benefitted from neo-adjuvant chemotherapy [11]. Currently, there is no reliable method with which to accurately stratify patients into surgical cytoreduction, preventing an individualized approach. A novel imaging-based prognostic signature, called the radiomic–prognostic–vector (RPV), based on conventional, contrast-enhanced CT, reliably identified the 5% of patients with a median overall survival (OS) of less than two years, independent of known prognostic factors [12]. Transcriptomic and proteomic analysis from two independent datasets showed a consistent correlation between RPV and stromal phenotype, a known poor prognostic indicator [12]. In a cohort of patients with primary colorectal cancer and liver metastases, the assessment of tumor-stroma interactions has been reported to independently predict survival and recurrence as a poor prognostic biomarker [13]. RPV could potentially identify women who are unlikely to benefit from conventional up-front cytoreductive surgery followed by chemotherapy [12].

The aim of this study was to evaluate whether this previously discovered CT-based RPV in women with high-grade serous ovarian cancer (HGSOC) patients, treated with up-front cytoreductive surgery, can be applied and validated at an external institution.

## Methods

### Study design and patients

This retrospective, observational cohort study was carried out with patients treated and operated upon at the Department of Gynecology, which is a European Society of Gynecologic Oncology (ESGO) Center of Excellence, and imaged at the Department of Biomedical Imaging and Image-guided Therapy both at the Medical University of Vienna (MUW), Austria, in collaboration with the Imperial College London [12]. A waiver of informed consent was granted and local Institutional Review Board approval for the retrospective analysis of human data was available (ESC 1919/2016). A local database was searched to identify women with primary HGSOC in whom a pre-treatment contrast-enhanced CT scan of the abdomen and pelvis was available between May 2002 and October 2017, to simulate dates from the discovery dataset [12]. Patients were included if there was histopathologic confirmation of HGSOC and at least one visible primary ovarian tumor mass equal to or larger than 2 cm in diameter. Patients with a history of other malignancy, previous bilateral salpingo-oophorectomy, or previous resection of the ovarian mass, significant imaging artifacts, incomplete image data, non-contrast examination, or non-HGSOC histology were excluded. Clinical characteristics were collected, including age, performance status, the International Federation of Gynecology and Obstetrics (FIGO) stage, tumor grade, histology subtype, post-operative residual disease, primary chemotherapy response, pre- and post-treatment CA125, treatment regimen, platinum resistance, date of progression, disease progression event, and date of death.

### Imaging technique and ovarian primary segmentation

All contrast-enhanced CT examinations were performed in the standard supine position with arms lifted above the head and in the portal venous phase after a standard dose of intravenous contrast medium. Due to the retrospective study design and long-term study period, the CT examinations were carried out using different machines from different vendors with variable protocols.

CT examinations were reviewed in consensus by two experienced radiologists blinded to clinical outcomes (G.J.W., 7 years of experience; A.G.R., 19 years of experience) to identify the primary ovarian tumor mass and any exclusion criteria. Lesions were then manually segmented by the junior radiologist using ITK-SNAP [14], including the complete tubo-ovarian mass, with cystic and solid components. The major gonadal vascular pedicle, free ascites, and peritoneal implants were excluded from segmentations. Adjacent parts of the bowel were carefully excluded. If there was doubt about the nature of any tissue, it was excluded from the segmentation. Bilateral tumors were segmented separately if a definite plane of separation was possible, or, in the event of tumor confluence, the entire mass was segmented. The senior radiologist then checked and adjusted the original segmentation.

### Radiomic measurement and exchange of software application

The RPV was originally derived by Lu et al using in-house TexLAB software to generate the radiomic features in MATLAB at the Imperial College London [12]. The RPV is a weighted, scaled, and centered sum of four radiomic features that are strongly predictive of OS and progression-free survival (PFS) [12]. We reproduced the image analysis conditions used by Lu et al and used the native reconstructed CT volumes for feature generation [12].

The RPV was encoded in a lightweight software based on TexLAB, called TexLABrpv, which computes only the previously defined and selected set of features and then outputs the RPV and associated risk level (low, medium, and high) for each patient. The RPV software was licensed to the Computational Imaging Research Laboratory at the Department of Biomedical Imaging and Image-guided Therapy of the MUW, Austria, specifically to analyze the segmented CT examinations on-site as an independent external validation, using the same decision boundaries as the original paper. The RPV tool, together with its related configuration files, libraries, and dependencies that are required for it to run efficiently and bug-free in an outside computing environment was provided without modification and in its original form by running in a containerized radiomic pipeline. Such containerization allows an easy deployment of the whole software chain across different computing environments and also different imaging centers.

If there were two masses in a patient, both masses were analyzed separately for RPV, and the highest RPV lesion was used for the per-patient analysis.

### Stromal tissue assessment

To validate the former discovery of an association between a higher stromal component and higher RPV values [12], we performed stromal fibronectin immunohistochemistry staining of RPV-high and RPV-low lesions in a subgroup of patients. The local tissue databank was retrospectively searched, and tissue specimens retrieved all lesions rated as RPV-high, as well as the equivalent number of the lowest RPV lesions, and additionally, a random selection of three groups each with the same number (*n* = 9 × 3, total additionally cases *n* = 27) or low RPV rated cases that had tissue available, with a total of 36 low RPV cases evaluated. Representative tumor samples of these specimens were stained using a fibronectin antibody (Ventana) as recommended by the manufacturer and evaluated for the fibronectin-expressing stromal component. A board-certified specialized gynecological pathologist performed a qualitative assessment of the percentage of stromal tissue content of each lesion in random order. The fraction of tumor stromal content was visually estimated, with the use of several other routine diagnostic procedures, such as tumor-infiltrating lymphocyte evaluation [15] of (PD-L1) assessment by immune score [16]. The reader was blinded to the original histopathological and RPV results.

### Statistical analysis

Statistical analysis was performed using R3.3.1 and MedCalc version 15.4. For patient age, tumor stage, residual disease, relapsed disease, and deceased a chi-square test was used. To assess the prognostic and predictive potential of the RPV score, follow-up, PFS, and OS the Cox proportional hazards model and the Kaplan–Meier analysis with a log-rank test were used. In addition, to understand differences in segmentations and possible output changes of RPV, an intraclass correlation coefficient (ICC) and Cohen’s kappa with Pearson and Spearman correlations, as well as a Dice statistic to compare segmentations between the junior and senior radiologists, were calculated. A *p*-value equal to or below 0.05 was regarded as statistically significant.

## Results

### Patient characteristics

**Fig. 1:**
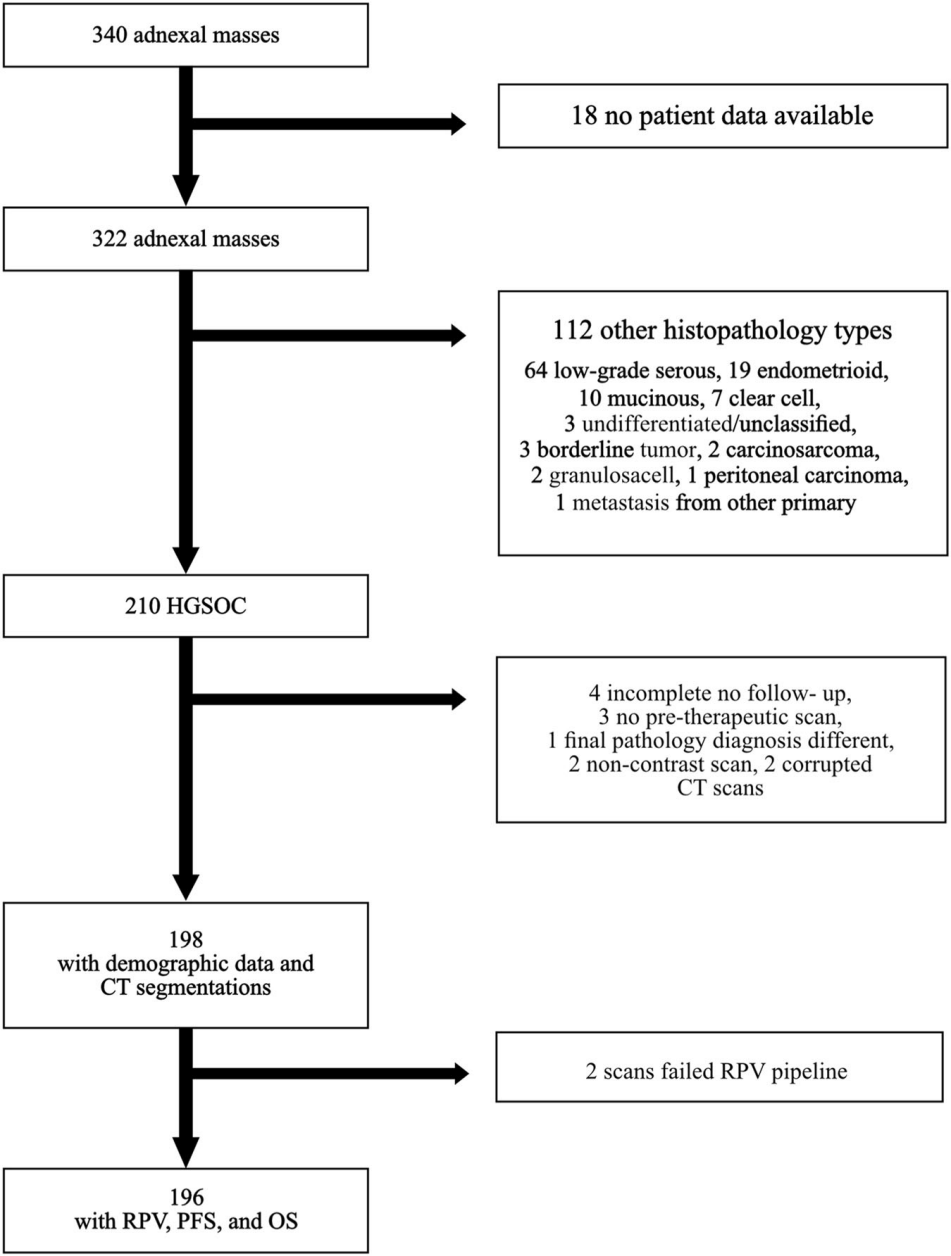
Flowchart of the final MUW HGSOC patients from the database search between May 2022 and October 2017 at the Department of Biomedical Imaging and Image-guided Therapy of the MUW. HGSOC, high-grade serous ovarian cancer; MUW, Medical University of Vienna external validation cohort

The final study cohort for the external validation of the RPV comprised 198 patients with a total of 244 HGSOC lesions with available imaging data at the Department of Biomedical Imaging and Image-guided Therapy of the MUW, Austria, within a time period between May 2002 and October 2017 (age range, 34–92 years; mean age, 59.8 years). Figure 1 shows the flow of patients from the initial database search to the final study cohort for the external validation of RPV. The majority of HGSOC lesions were bilateral (*N* = 97), followed by right-sided (*N* = 54) and left-sided (*N* = 46) lesions, with a median lesion size of 11.28 mm (size range, 2.48–22.75 mm) on CT imaging. For bilateral disease, 46 patients presented with separate right- and left-sided HGSOC lesions. To compare the MUW cohort with the discovery dataset from the Hammersmith Hospital (HH), Imperial College Healthcare NHS Trust, London, which was presented in [12], patient demographic data were analyzed. Table 1 shows a good comparison of the validation and discovery datasets for age, lesion stage, complete gross resection (CGR) of disease at primary surgery, patient follow-up, and OS. There was a shorter PFS in the MUW cohort, but this did not reach statistical significance (*p* = 0.135, Supplementary Fig. S1). There was a larger number of patients with disease relapse during the period of follow-up in the MUW cohort (64.1%) compared to the HH cohort (48.2%), *p* = 0.00025, although relapse status was not known in 20 cases (8.9%) of the HH cohort. Further clinical data of the MUW cohort can be found in Supplementary Table S1.

**Table 1:** Comparison of patient demographic data from the MUW, Austria, and HH, Imperial College Healthcare NHS Trust, London cohorts

Characteristics		MUW (*n* = 198)	HH (*n* = 224)	*p*-value
Age, (%)	< 60	102 (51.5)	97 (43.4)	ns
	> 60	96 (48.5)	127 (56.7)	
Stage, (%)	I	6 (3)	9 (4)	ns
	II	13 (6.5)	16 (7.1)	
	III	156 (78.8)	135 (60.3)	
	IV	23 (11.6)	61 (27.2)	
	Unknown	–	3 (1.3)	
Residual disease, (%)	CGR	98 (49.5)	106 (47.3)	0.0131
	Non-CGR	79 (39.9)	47 (21)	
	Unknown	21 (10.6)	71 (31.7)	
Follow-up, (months)	Median	57.6	49.3	ns
	IQR	31.6–95.6	16.5–71.8	
PFS, (months)	Median	18.1	21.1	ns
	IQR	11.8–32.8	11.7–50.9	
OS, (months)	Median	48.69	51.8	ns
	IQR	27.0–na	25.5–na	
Relapsed, (%)	No	71 (35.9)	96 (42.9)	6.903 × 10^−^^6^
	Yes	127 (64.1)	108 (48.2)	
	Unknown	–	20 (8.9)	
Deceased, (%)	No	102 (52)	133 (59.4)	ns
	Yes	96 (48)	90 (40.2)	
	Unknown	–	1 (0.4)	

*CGR* complete gross resection, *PFS* progression-free survival, *OS* overall survival, *IQR* interquartile range, *MUW* Medical University of Vienna validation cohort, *HH* Hammersmith Hospital, London cohort

### External validation of the RPV

Among the 244 lesions (196 patients) in the MUW cohort, 185 adnexal lesions (147 patients) were classified as RPV-low (75%), 50 lesions (40 patients) were classified as RPV-medium (20%), and nine lesions (in nine patients) were classified as RPV-high (5%).

We first analyzed the association between RPV in the MUW cohort with Cox regression in 196 patients with both RPV and OS data available. Univariate analysis demonstrated that OS was significantly associated with FIGO stage (III/IV vs I/II), postoperative residual disease (no macroscopic residual disease vs any), and age (< 60 vs > 60 years), and RPV demonstrated a significant association with OS when combining RPV-low and RPV-medium in contrast to RPV-high (Table 2). In the multivariate Cox analysis, an RPV-high retained its significant association with OS (HR 3.17, 95% CI: 1.32–7.60, *p* = 0.0099), as did FIGO stage and post-operative residual disease, although age was no longer associated.

**Table 2:** OS Cox regression analysis of the MUW cohort, with RPV high vs low/medium

	Univariate		Multivariate	
Feature	HR (95% CI)	*p*-value	HR (95% CI)	*p*-value
RPV (high vs others)	2.56 (1.11–5.87)	0.0268	3.17 (1.32–7.60)	0.00991
FIGO stage (I/II vs III/IV)	1.91 (1.31–2.78)	0.000852	1.79 (1.11–2.86)	0.016
Residual disease	2.33 (1.51–3.60)	0.000132	2.04 (1.30–3.20)	0.00204
Age (< 60 vs > 60)	1.56 (1.04–2.34)	0.0318	1.42 (0.91–2.21)	0.121

*RPV* radiomic prognostic vector, *OS* overall survival, *HR* hazard ratio, *CI* confidence interval, *MUW* Medical University of Vienna validation cohort, *FIGO* International Federation of Gynecology and Obstetrics

**Fig. 2:**
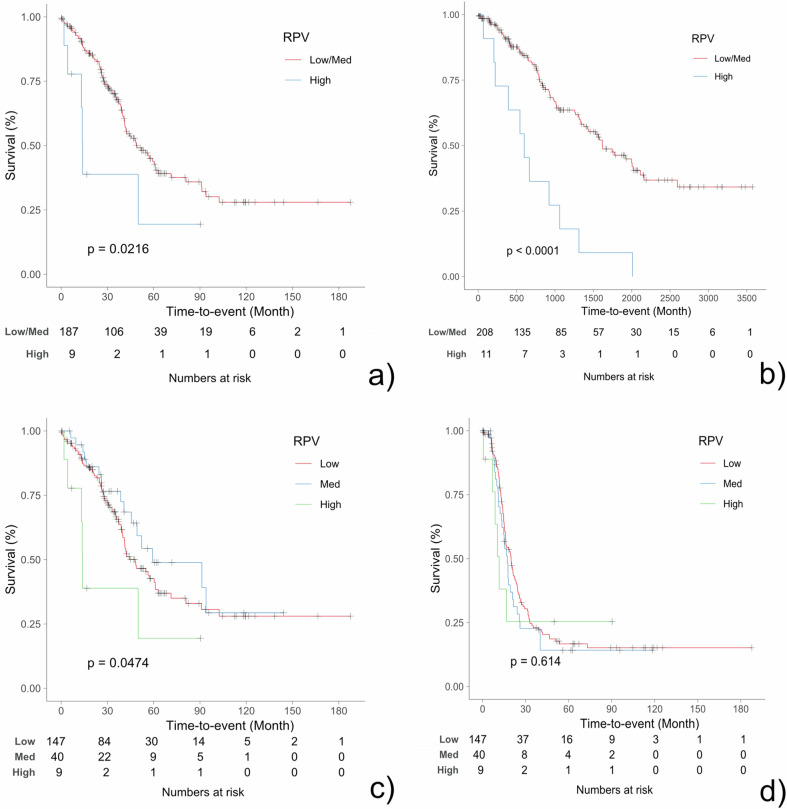
Kaplan–Meier analysis revealed a significant difference in OS between combined low- or medium-RPV patients compared to patients with high-RPV (**a**), similar to the discovery cohort HH (**b**). A significant difference in OS was found when categorizing MUW patients separately as RPV-low, -medium, or -high (**c**). There was no significant difference in PFS for separate categories (**d**). RPV, radiomic prognostic vector; OS, overall survival; PFS, progression-free survival

Kaplan–Meier survival analysis (Fig. 2a, b) demonstrated a significant difference in OS between RPV-low or RPV-medium patients combined (*n* = 187) and those with RPV-high (*n* = 9), *p* = 0.0216, similar to the discovery HH dataset. There was no statistically significant difference in PFS between these groups.

When categorizing patients separately as RPV-low (*n* = 147), RPV-medium (*n* = 40), or RPV-high (*n* = 9), OS was significantly different between the groups (*p* = 0.047), but there was no significant difference in PFS (Fig. 2c, d).

### Tumor biological features linked to RPV

**Fig. 3:**
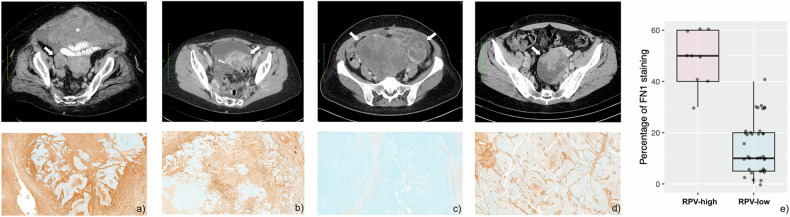
Fibronectin staining for stromal content. Example of four different patients with a corresponding CT image example of the adnexal lesion demonstrating immunohistochemical expression of fibronectin in high-risk RPV (**a**, **b**) compared to low-risk RPV (**c**, **d**). High-risk RPV cases showed a higher proportion of tumor-associated stroma (brown color) with lower tumor cellularity (blue color) compared to low-risk cases. Boxplots of the percentage of fibronectin staining in a sub-set of high-risk and low-risk RPV-scored lesions (**e**). The nine cases with high-risk RPV values, nine cases with the lowest-risk RPV values, and a random sample of a total of 27 cases assessed as low-risk RPV were evaluated

Subgroup analysis of stromal content was undertaken in the nine cases with RPV-high lesions compared to nine cases with RPV-low lesions, and three groups of randomly selected RPV-low lesions with a total of 27 cases. Fibronectin staining revealed a high proportion of tumor-associated stroma in the RPV-high group (Fig. 3). We found that RPV-high was positively correlated with the expression of a stromal marker, fibronectin, and therefore, higher proportions of stromal content (mean 48.9%; SD 10.5) compared to the RPV-low subgroup (mean 14.9%; SD 10.5), respectively (*p*-value = 7.119e-06 by Wilcox test). More details of the subgroup analysis can be found in Supplementary Fig. S2.

### Difference in RPV output between the HH cohort and the Junior and corrected senior annotations

**Fig. 4:**
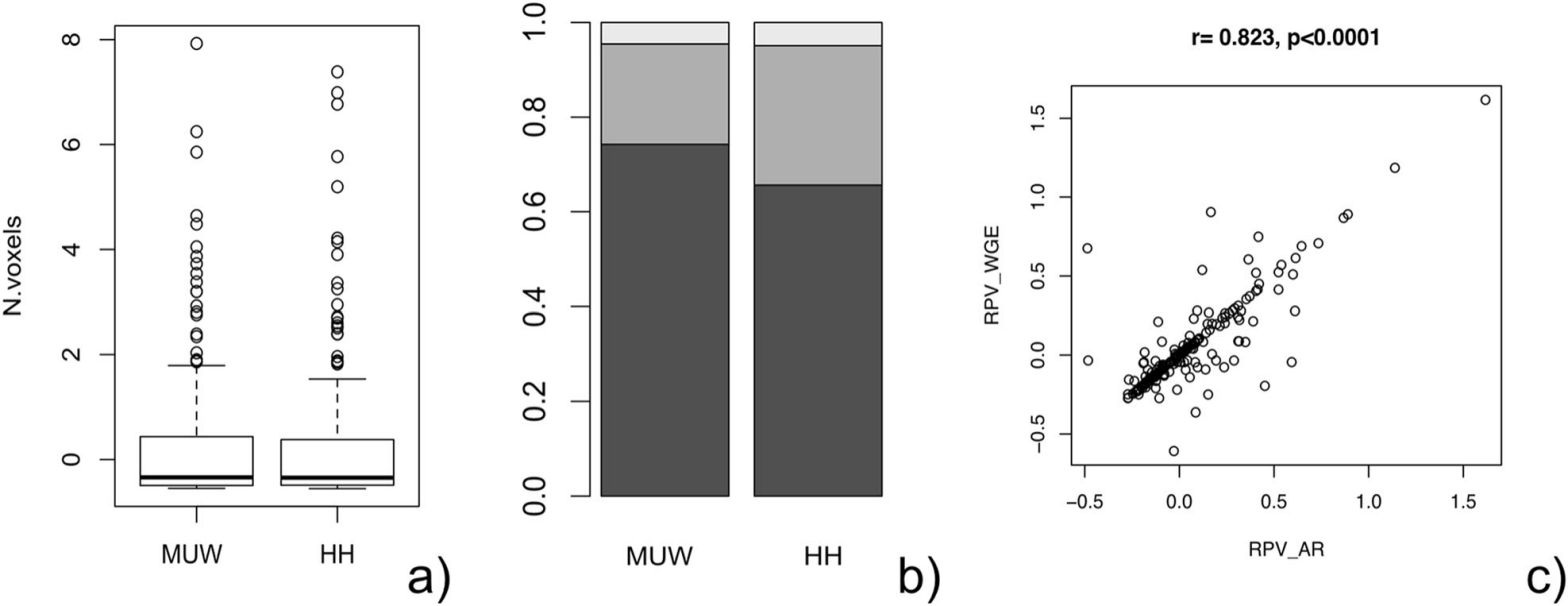
Comparison of radiomics between MUW (*n* = 196) and HH (*n* = 219) and between observers. Volume of segmented masses based on voxels between MUW and HH (**a**). RPV distribution between MUW and HH as a histogram (**b**). RPV correlation between junior and final corrected senior segmentations, MUW cohort (**c**). RPV, radiomic prognostic vector; MUW, Medical University of Vienna external validation cohort; HH, Hammersmith Hospital, London cohort

The volume of segmented masses and the proportion of RPV-low, -medium, and -high cases between the HH discovery set and the MUW cohorts were similar (Fig. 4a, b).

The ICC between junior and final corrected senior annotations on the RPV output revealed an 82% reliability for the output, an ICC = 0.8231, with a Pearson correlation coefficient of *r* = 0.8225 (*p* < 0.001, 95% CI: 0.771–0.863) (Fig. 4c).

With regard to investigating the difference between the segmentations of the junior radiologist and the final adjusted expert segmentations, all 244 segmentations were analyzed. For comparison of RPV, we expressed RPV as ordinal (low, medium, and high) and as metric (RPV range −0.608 to 1.617) units.

For Cohen’s kappa agreement between senior and junior segmentations in 244 lesions, the RPV output demonstrated a good agreement, with *k* = 0.767, SE = 0.063, and a Spearman correlation coefficient of rho = 0.783 (*p* < 0.0001, 95% CI: 0.722–0.832).

With regard to the comparison of both sets of annotations on a volume level, a quantitative Sorensen–Dice coefficient (DSC) was calculated. On the volume level, both sets of annotations showed a similarity of 91% (DSC = 0.91, 95% CI: 0.884–0.931). The results are summarized in Table 3. In addition, Table 4 summarizes quantitative differences between both sets of annotations adjusted by the senior radiologist, which were subdivided into DSC ranges. No change was made by a senior radiologist in 50 cases (*N* = 50, 25.25%); however, the target lesion was missed in one case (*N* = 1, 0.51%) by the junior radiologist. The inclusion of a myomatous uterus into the segmentation was observed in three cases (*N* = 3, 1.52%), whereas the omission of solid or cystic-solid tumor components was present in seven cases (*N* = 7, 3.54%). For the vast majority of 137 cases, only small-to-moderate changes had to be made by the senior radiologist (*N* = 137, 69.19%).

**Table 3:** Summary of agreement, reliability, and similarity of the RPV output between junior radiologists’ annotations and senior radiologists corrected annotations in MUW HGSOC lesions

Kappa		ICC		DSC	
k	0.767	ICC	0.8231	DSC	0.910
SE	0.063	95% CI	0.772–0.864	95% CI	0.884–0.931
Spearman	0.783	Pearson	0.8225	SE	0.012
*p*-value	< 0.0001	*p*-value	< 0.0001		
95% CI	0.722–0.832	95% CI	0.771–0.863		

In cases with bilateral ovarian masses, both masses were included in the analysis, with a final single RPV output per patient

*k* weighted kappa, *ICC* intraclass correlation coefficient, *DSC* Dice coefficient, *SE* standard error, *CI* confidence interval, *MUW* Medical University of Vienna validation cohort, *HGSOC* high-grade serous ovarian cancer

**Table 4:** Summary of differences between senior and junior radiologists’ segmentations adjusted for the senior radiologist in MUW HGSOC lesions for DSC ranges

DSC range	*N*	(%)	Reason for difference	*N*
0	1/198	(0.51)	Segmentation, not target lesion	1
0.13–0.15	2/198	(1.01)	Missed cystic/solid component	2
0.23–0.27	2/198	(1.01)	Myomatous uterus included	1
			Missed cystic/solid tissue component	1
0.30	1/198	(0.51)	Segmentation smaller	1
0.46	1/198	(0.51)	Segmentation smaller	1
0.52–0.57	4/198	(2.02)	Missed solid component	2
			Segmentation smaller	1
			Segmentation larger	1
0.60–0.69	5/198	(2.53)	Segmentation smaller	5
0.70–0.79	12/198	(6.06)	Segmentation is a little smaller	5
			Segmentation is a little larger	5
			Missed solid tissue component	1
			Small part of the small bowel included	1
0.80–0.89	25/198	(12.63)	Segmentation is a little smaller	16
			Segmentation is a little larger	8
			Missed solid tissue component	1
0.90–0.99	95/198	(47.98)	Segmentation is a little smaller	70
			Segmentation is a little larger	23
			Myomatous uterus included	2
1	50/198	(25.25)	No changes	50

*DSC* Dice coefficient, *MUW* Medical University of Vienna validation cohort

## Discussion

In advanced OC, risk stratification to optimize the patients’ initial treatment pathway remains challenging. Patient selection for up-front radical cytoreduction warrants the definition of better algorithms to identify candidates who would most benefit from surgery, while avoiding surgery in those unlikely to benefit. In this external validation study, undertaken by licensing radiomic signature (RPV) software to an external institution, we confirmed that patients with HGSOC classified as RPV-high had a three times worse OS when compared to patients classified as RPV-low or -medium (HR 3.17, 95% CI: 1.32–7.60, *p* = 0.0099) after adjusting for known clinicopathological prognostic factors such as FIGO-stage, postoperative residual disease, and age. Kaplan–Meier survival analysis confirmed a significantly shorter OS (*p* = 0.0216) in the RPV-high patients. These findings support RPV as an objective pre-treatment clinical decision tool, by identifying the 5% of patients with significantly worse OS, as per the discovery cohort, regardless of other prognostic factors [12]. Furthermore, we found a similar biological interpretation of RPV, with a significantly higher percentage of stromal content in RPV-high cases compared to a sub-set of RPV-low cases, based on immunohistochemical fibronectin expression (*p* < 0.0001), which confirmed a stroma-rich phenotype in RPV-high cases. The RPV score was not associated with PFS in this cohort. This could be related to the higher proportion of women who had relapsed in the study cohort compared to the discovery cohort, with a trend toward shorter PFS.

Lu et al reported an association of the stromal marker fibronectin with RPV-high lesions, providing a biological basis for the RPV [12]. Higher fibronectin and stromal content have been reported to be highly correlated with shorter OS in patients with OC [17–19]. High fibronectin expression was an independent prognostic factor for worse OS and PFS in a study of 1512 breast cancer patients [20]. A similar association between poor prognosis and high expression of fibronectin was also reported in esophageal squamous cell carcinoma patients [21]. The finding of this association in other tumor types raises the possibility that RPV may be more widely generalized. The activation of a stroma-rich phenotype and DNA damage response may be advantageous for new drug development and alternative targeted therapeutic agents in the future [12].

In their original paper, Lu et al included an internal and an open-source validation data set [12]. In the first attempt to externally validate the RPV score in patients from another institution, CT scans from women treated in an ESGO-accredited center in Germany were analyzed in the same lab in which the RPV was developed [22]. The study confirmed that RPV-high cases were associated with a significantly worse PFS, identifying those women who relapsed early despite complete macroscopic tumor clearance at surgery. However, there was no significant association with OS [22]. The study did not undertake to confirm the association with a stroma-rich phenotype. Despite some differences between the exact findings in the original study and the previous and current validation studies, all three have demonstrated poor patient outcomes in the RPV-high group, strengthening the evidence that RPV provides robust risk stratification. External validation of radiomics models has been reported in other tumor types and these studies have used publicly available open-source data [23] or independent external imaging datasets from other centers [24–28]. These studies lacked information about whether the images or the software were exchanged for validation. Nevertheless, validating the discovered radiomics model with an independent sample of patients with at least 100 cases [29] is considered to be the next step toward translation into clinical practice.

The primary focus of the current study was on replicating the original approach by transmitting the RPV tool to a different institutional environment. We have shown the feasibility of this approach. We did not perform any additional pre-processing in order to replicate and maintain uniformity with the original discovery study, representing one possible limitation of our study [12]. To address this, future analysis will focus on the impact of pre-processing techniques, and thus, identify potential improvements to confirm the generalizability of the RPV. In addition, blinded inter-reader assessment of junior and senior annotations to unveil any variability in the RPV output, and comparison to an automated segmentation tool for adnexal lesions, will need to be evaluated. Histological analysis was possible in only a small subset of cases and this work should be extended in future studies. Nonetheless, this validation of the histological findings underpins the biological basis of the RPV and is a huge strength of this study.

We have investigated the ability of a CT-based RPV to identify HGSOC patients at risk of limited OS, using standard-of-care pre-treatment imaging. The biological interpretation of RPV-high, a stroma-rich histological sub-type, was also found. This retrospective external validation is an essential step to ensure safe and generalizable translation, which will now be followed by prospective testing. The RPV software as a medical device could be used in the future as an objective clinical decision support tool, with the intended use of identifying those patients who may benefit from initial neo-adjuvant chemotherapy as an alternative to up-front surgery, thus offering a personalized approach to treatment in HGSOC patients in a poor prognostic group.

## Supplementary information

Supplementary Material

## Abbreviations

CGR: Complete gross resection
DSC: Sorensen–Dice coefficient
ESGO: European Society of Gynecologic Oncology
FIGO: International Federation of Gynecology and Obstetrics
HGSOC: High-grade serous ovarian cancer
HH: Hammersmith hospital
IQR: Interquartile range
MUW: Medical University of Vienna
OS: Overall survival
PFS: Progression free survival
RPV: Radiomic prognostic vector
