# Supplementary material for: CT-based radiomic prognostic vector (RPV) predicts survival and stromal histology in high-grade serous ovarian cancer: an external validation study
Source: Eur Radiol. 2024 Dec 11;35(6):3110–9. doi: 10.1007/s00330-024-11267-5 (PMC12081573; doi:10.1007/s00330-024-11267-5)

Supplementary Material

Supplementary Table S1: Summary of descriptive characteristics of the MUW external validation cohort

CR – complete response, PR – partial response, SD – stable disease, PD – progressive disease, PARPi – poly (ADP-ribose) polymerase inhibitor, MUW –Medical University of Vienna validation cohort, CT – computer tomography, FIGO – International Federation of Gynaecology and Obstetrics, rt – right side, lt – left side.

**Characteristics**		**N**	
**Vendor of CT**	Siemens	158	(79.80 %)
	Philips	30	(15.15 %)
	Toshiba	9	(4.55 %)
	GE	1	(0.51 %)
**Post-OP residual disease**	CGR	98	(49.49 %)
	Non-CGR, < 2cm	41	(20.71 %)
	Non-CGR, >2cm	38	(19.19 %)
	Unknown	21	(10.61 %)
**FIGO stage**	1B	1	(0.51 %)
	1C	5	(2.53 %)
	2A	1	(0.51%)
	2B	7	(3.54 %)
	2C	5	(2.53 %)
	3A	9	(4.55 %)
	3B	15	(7.58 %)
	3C	132	(66.67 %)
	4A	5	((2.53 %)
	4B	18	(9.09 %)
**Grade**	1	2	(1.01 %)
	2	2	(1.01 %)
	3	194	(97.98%)
**Chemotherapy**	Yes	181	(91.41 %)
	No	17	(8.59 %)
**Platinum-based chemotherapy**	Yes	181	(91.41 %)
	No	2	(1.01 %)
	Unknown	15	(7.58 %)
**Response to treatment**	CR	145	(73.23 %)
	PR	23	(11.62 %)
	SD	1	(0.51 %)
	PD	13	(6.57 %)
	Unknown	16	(8.08 %)
**Relapse disease**	Yes	127	(64.14 %)
	No	71	(35.86 %)
**Therapy after relapse**	Chemotherapy	92	(72.44 %)

	Hemotherapy + PARPi	10	(7.87 %)
	Surgery + Chemotherapy	10	(7.87 %)
	Irradiation	2	(1.57 %)
	Missing	85	(66.93 %)
**RPV output (per lesion, n=244)**	Low	185 (rt 157; lt 28)	(75.82 %)
	Medium	50 (rt 36; lt 14)	(20.49 %)
	High	9 (rt 5; lt4)	(3.69 %)
**RPV output in bilateral lesions (n=46 patients)**	Bilateral low (per lesion)	57 (rt 29; lt 28)	(61.96%)
	Bilateral medium (per lesion)	29 (rt 15; lt 14)	(31.52%)
	Bilateral high (per lesion)	6 (rt 2; lt 4)	(6.52%)
	Bilateral low (per RPV)	19	(41.30%)
	Bilateral medium (per RPV)	6	(13.04%)
	Bilateral high (per RPV)	-	-

Supplementary Table S2: Steps undertaken for the radiomic analysis.

1	We used the IBSI CT phantom.There are no reference values on the IBSI website, (from http://theibsi.github.io/ibsi1/) based on the CT phantom
2	We therefore used Pyradiomics as this is an IBSI compliant radiomic tool. This produced 112 features that could be used for the analysis
3	TexLAB v2 produced 666 features using one grey level, but by selecting all grey levels, there were 3906 features
4	42 features were identical in name and output. 4 features were identical in name and within 6% absolute difference
5	The following is true of features that make up the RPV vector:<br>- FOS_Imedian (exact match with pyradiomics)<br>- NGTDM_Contra_25HUgl (within 2% difference)<br>- GLRLM_SRLGLE_25HUgl (exact match with pyradiomics)<br>- FD_max_25HUgl (feature is not supportet by pyradiomics)

Supplementary Figure S1: Kaplan-Meier analysis of patient survival comparing MUW cohort with HH cohort. Progression-free survival (a) and overall survival (b). The progression-free survival at MUW showed a trend suggesting shorter PFS, but this was not statistically significant. OS was equivalent in the two cohorts.

OS – overall survival, PFS – progression free survival, MUW – Medical University of Vienna external validation cohort, HH – Hammersmith Hospital, London cohort.

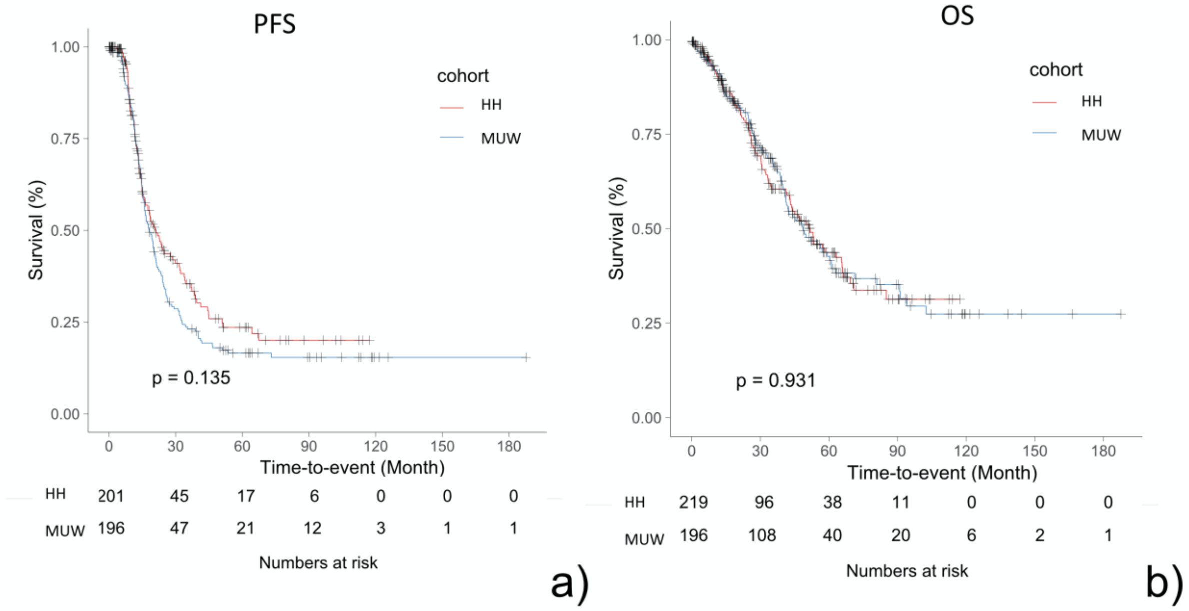

Supplementary Figure S2: Boxplots illustrating the subgroup analysis of stromal content of the nine RPV-high and RPV-low lesions. For RPV-low lesions subgroups with the nine lowest calculated RPV values (RPV-lowest), and three groups (group 1-3) with a random selection of nine RPV-low calculated cases were analysed, respectively. The graph depicts the positive correlation of RPV-high with the stromal marker, fibronectin, in each subcategory. P-value = 5.869e-05 by Kruskal Wallis test.

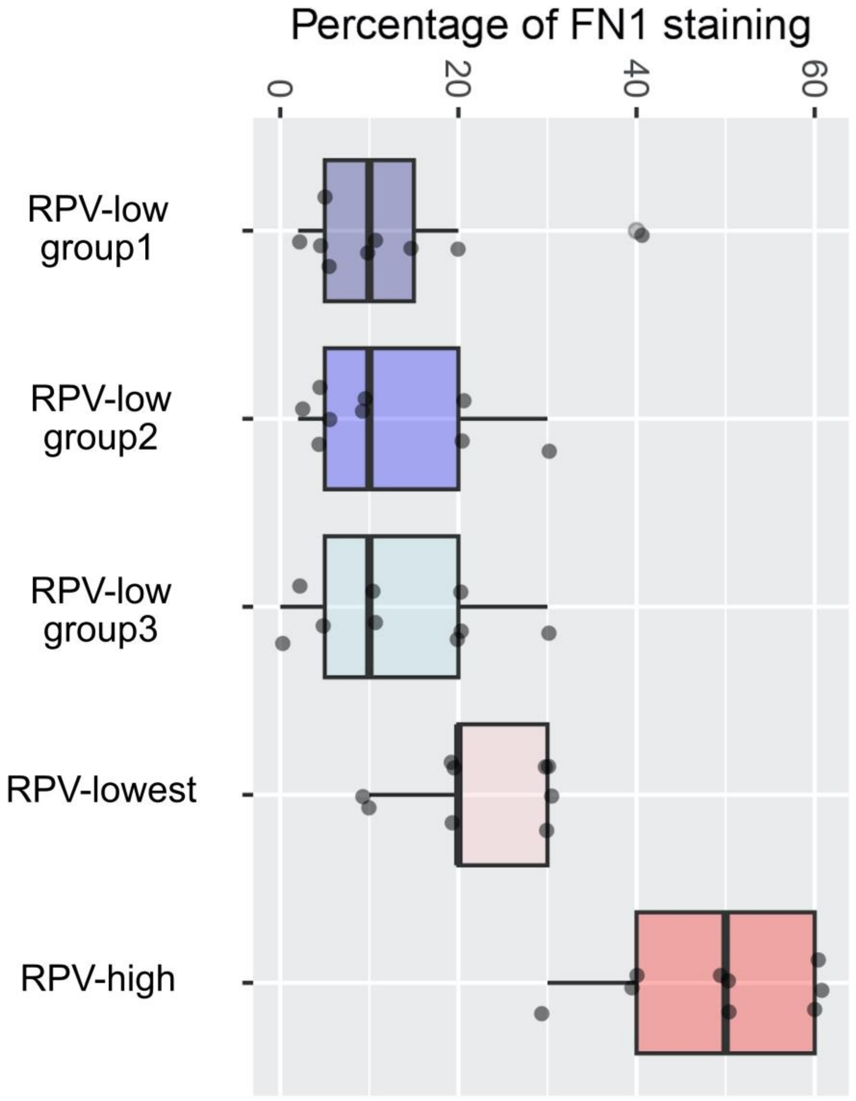

Supplement: Supplementary file 1 — Supplementary Material [file 330_2024_11267_MOESM1_ESM.pdf]
